# Nanomechanics of graphene oxide-bacteriophage based self-assembled porous composites

**DOI:** 10.1038/s41598-020-72372-1

**Published:** 2020-09-24

**Authors:** Yiwei Sun, Paolo Passaretti, Ignacio Hernandez, Jesus Gonzalez, Wei Liu, Fernando Rodriguez, David J. Dunstan, Pola Goldberg Oppenheimer, Colin J. Humphreys

**Affiliations:** 1 Queen Mary University of London, School of Engineering and Materials Science, London, E1 4NS UK; 2 Institute of Cancer and Genomic Science, University of Birmingham, Birmingham, B15 2TT UK; 3 Departamento CITIMAC, Universidad de Cantabria, Santander, 39005 Spain; 4 College of Information Science and Electronic Engineering, Zhejiang University, Hangzhou, 310027 China; 5 School of Physics and Astronomy, Queen Mary University of London, London, E1 4NS UK; 6 School of Chemical Engineering, University of Birmingham, Birmingham, B15 2TT UK

**Keywords:** Biophysics, Materials science, Nanoscience and technology

## Abstract

Graphene oxide, integrated with the filamentous bacteriophage M13, forms a 3D large-scale multifunctional porous structure by self-assembly, with considerable potential for applications. We performed Raman spectroscopy under pressure on this porous composite to understand its fundamental mechanics. The results show that at low applied pressure, the $sp^2$ bonds of graphene oxide stiffen very little with increasing pressure, suggesting a complicated behaviour of water intercalated between the graphene layers. The key message of this paper is that water in a confined space can have a significant impact on the nanostructure that hosts it. We introduced carbon nanotubes during the self-assembly of graphene oxide and M13, and a similar porous macro-structure was observed. However, in the presence of carbon nanotubes, pressure is transmitted to the $sp^2$ bonds of graphene oxide straightforwardly as in graphite. The electrical conductivity of the composite containing carbon nanotubes is improved by about 30 times at a bias voltage of 10 V. This observation suggests that the porous structure has potential in applications where good electrical conductivity is desired, such as sensors and batteries.

## Introduction

Graphene oxide (GO) is graphene with oxygen functional groups, which are commonly hydroxyl, carboxyl and carbonyl^1^. GO is hydrophilic and water can be easily intercalated between GO layers^2^. It can form porous structures by self-assembly, of low density and large surface area^3^, which are promising for applications such as energy storage^4^. However, GO is usually much less electrically conductive than graphene^3^. GO-based structures would be more appealing for applications like sensors, catalysts, energy conversion, batteries and supercapacitors, where this porous structure could be employed if its electrical conductivity can be improved^3^.

Previously, we reported a novel, efficient and scalable strategy to assemble GO into three-dimensional (3D) porous sponges^5^ by exploiting the filamentous bacteriophage M13^6^. We named the sponge *Graphage*13. It is multifunctional due to the possibility to functionalise both graphene and M13^5^. In particular, M13 is very well known for techniques like phage display and other chemical modifications, which allow the bacteriophage to display on its surface functional chemical groups, nanoparticles, enzymes, proteins, small peptides, DNA and antibodies^7^. Therefore, depending on the functionalisation, the structure can acquire a specific ability such as performing chemical reactions, binding molecules, etc^3^. In general, the ability of bacteriophages of selective binding and their versatility in genetic modification result in a large variety of bacteriophage-based structures, the properties of which, such as electrical conductivity, can be very different^8^. For example, a water-soluble conductive polyaniline-M13 composite can be assembled with M13 as a template^9^. Biological reactions can further modify the electrical conductivity of bacteriophage-based structures^10^. Our method used here is convenient and environmentally-friendly as it does not require conditions such as high pressure, temperature or extreme pH values, that are usually required for the production of similar GO sponges^3,11–14^.

The very stiff in-plane $sp^2$-bonds and the nanoscale layered structure of GO make it mechanically similar to the well-known graphite. The frequency of the graphite mode (G-mode, C–C stretching)^15^ of the GO can be measured routinely by Raman spectroscopy under pressure, quantifying the bond stiffness and anharmonicity. The results contribute to a comprehensive understanding of the fundamental mechanics of these composites.

The shift of the G-mode frequency of graphite with pressure was measured by Hanfland et al.^16^. The initial shift rate is 4.7 cm^−1^ GPa^−1^ with a notable sublinear curvature^16^. We have an interpretation for this curvature, summarised as follows—the small out-of-plane stiffness (along the *c*-axis) of graphite results in a large compressive strain along the *c*-axis under hydrostatic pressure. The in-plane force, the product of pressure and area (normal to the much compressed graphite planes), is therefore reduced below a linear dependence on pressure. The in-plane phonon frequency, shifting nearly linearly with the in-plane deformation^17^, therefore shifts strongly sublinearly with pressure.

The pressure coefficient of the G-mode of GO was reported as 3.8 cm^−1^ GPa^−1^ using a linear fit over the range 0–8 GPa^18^. Compared to graphite, GO has an even softer interlayer interaction and therefore its G-mode frequency should shift even more sublinearly with pressure. Not surprisingly, a linear fit to sub-linear data does give a smaller initial shift rate. Besides stiffening the $sp^2$-bonds, high pressure can also induce the re-distribution of functional groups on the GO surface^19^ and insert molecules like Ar, N$_2$, NH$_3$ and water (in other word, small molecules that are available in the environment) between the GO layers^20^. These become relevant, when we look for interpretation of the unexpected results reported in this paper.

**Figure 1:**
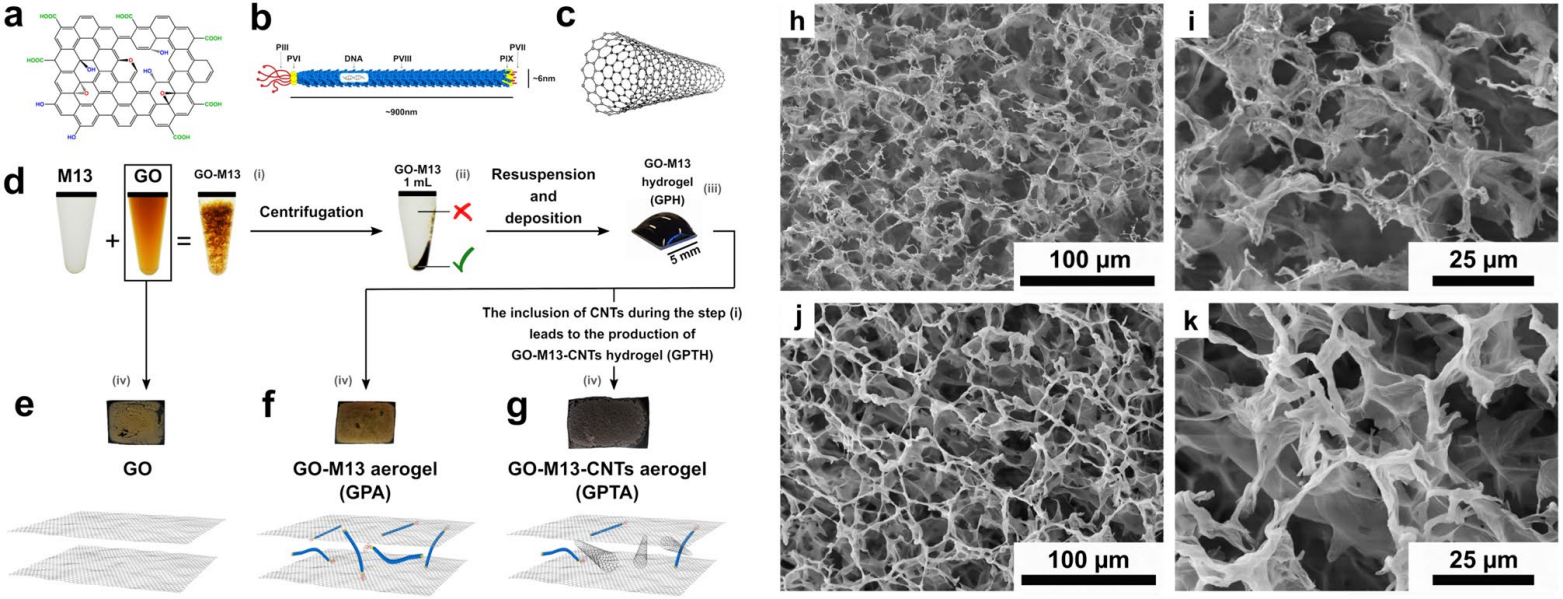
Schematics of (**a**) graphene oxide, (**b**) M13 bacteriophage and (**c**) carbon nanotubes. (**d**) Overview of the fabrication process of GraPhage13 aerogels including the four main steps: (i) assembly, (ii) precipitation, (iii) deposition and (iv) drying^5^. Images of the aerogels of (**e**) GO, (**f**) GO-M13 and (**g**) GO-M13-CNT. (**h**)–(**k**) are the secondary electron SEM images of GO-M13-CNT at two different magnifications in two random areas.

This work analyses the Raman spectra of GO and GO-M13 composites under pressure, especially over the low-pressure range 0–5 GPa. The pressure was calibrated by the ruby R1 line^21^ and the uncertainties from non-hydrostaticity were monitored by ruby spheres in different locations. We introduced carbon nanotubes (CNTs) during the self-assembly of GO-M13, aiming to improve the mechanical stiffness and stability of the composite. We also obtained a much improved electrical conductivity of the composite after introducing CNTs, which enables the high potential of GO porous structure to be achieved in applications where good electrical conductivity is desired. Nanoscale schematics, images and the fabrication process of the three composites (GO, GO-M13 and GO-M13-CNT) studied in this work are shown in Fig. 1a–k.

## Experimental

We followed our previous route to prepare the GO-M13 sample as a hydrogel^5^. In brief, we mixed 0.3 mg GO and 0.3 mg M13 in 1 mL deionized water. We then placed the microtube on an orbital shaker at 250 rpm for 15 min. After centrifuging it at 15,000 rpm (SciSpin MICRO Centrifuge, RCF = 15,100*g*) for 1 min, we removed 0.9 mL of the supernatant and then sonicated the pellet for 5 min. The weight ratio 1:1 of the GO and M13 after a 5 min sonication are demonstrated in Ref.^5^ to be the optimized recipe for the complete formation of the hydrogel with neither GO nor M13 remaining in the supernatant. We prepared pure GO pellets and GO-M13-CNT composite pellets by the same procedure, with no M13 in the pure GO, and 0.3 mg CNTs introduced before M13 for GO-M13-CNT. The CNT specimens used here were commercial CoMoCAT single-walled carbon nanotubes (SWCNTs) from Sigma-Aldrich. The product number of the sample is 704148. Its properties and characteristics are available on the product webpage. The tube diameter is 0.8 ± 0.1 nm. The mass percentage of carbon is over 90%, of SWCNTs is over 77% among carbon and of (6, 5) tubes is over 50% among SWCNTs. Furthermore, Brintlinger *et al.* used field-emission scanning electron microscopy as a probe of electrical connectivity of CNTs on insulator substrates^22^.

To apply high pressure, we loaded fragments of these prepared pellets in a membrane diamond anvil cell (anvil of 500 $\mu$m culet size and Type IIa diamonds). Water was used as the pressure transmitting medium (PTM) as the porous composite was formed in water in the synthesis routine. We used the ruby luminescence R1 line for pressure calibration^21^. Three ruby spheres (15 $\mu$m diameter) were loaded in the cell in different locations to monitor the hydrostaticity at high pressure.

Non-polarized Raman measurements were performed in the backscattering geometry with a Horiba T64000 Raman system (confocal microscope, single 1800 grooves/mm grating, 100 $\mu$m slit and a liquid N$_2$-cooled CCD detector (Jobin-Yvon Symphony)). An edge filter for the 647 nm laser line from a Coherent Innova Spectrum 70C Ar$^{+}$-Kr$^{+}$ laser was used. We kept the laser power on the GO sample below 5 mW to avoid significant laser-heating effects and the concomitant softening of the Raman peaks. We observed black aggregates (probably burnt M13) in GO-M13 and GO-M13-CNT at 5 mW and we reduced the laser power to 1 mW on the sample to avoid these.

Separately, to obtain the aerogel for scanning electron microscope (SEM) imaging, we deposited 50 $\mu$L of the hydrogel on a Si substrate cleaned by CO$_{2}$ snow jet on a hot plate at $250\,^{\circ }\hbox {C}$, and dried it for 1 h in a vacuum chamber at about 90 Pa. SEM images of GO-M13-CNT (Fig. 1h–k) show similar porous structures to GO-M13, the images of which are available in the Supplementary information (SI) and Ref.^5^.

To perform the I–V characterisation, the devices were prepared by coating the hydrogel on an oxide wafer with pre-patterned electrodes (Au/Cr 50 nm/5 nm; device area 500 $\mu$m $\times$ 500 $\mu$m defined by the electrodes), then baked at ${50}\,{^{\circ }}\hbox {C}$ (at which the M13 is stable^23^) for 10 min. The I–V curves were measured by a semiconductor analyzer (Agilent B1500) and a probe station.

## Results and discussions

### GO

**Figure 2:**
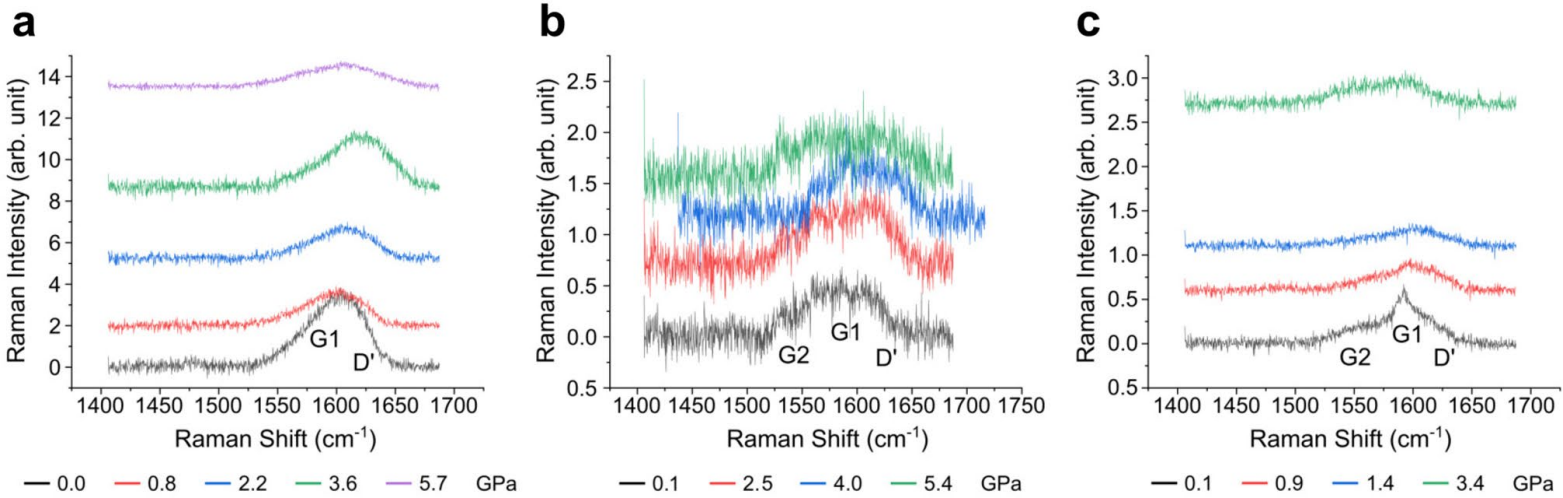
Raman spectra of (**a**) GO, (**b**) GO-M13 and (**c**) GO-M13-CNT over the G-mode frequency range. They are vertically shifted proportionally to the pressure at which they were obtained for clarity. The labels on the peaks are the interpretations made after fitting.

Figure 2a shows the Raman spectra over the G-mode range of GO at different pressures. For clarity, spectra are vertically shifted proportionally to the pressure at which they were obtained. The upshift of the Raman peaks with increasing pressure is clear except at 5.7 GPa, where the peak appears to downshift from the previous pressure point. The Raman profiles are broad and asymmetric, suggesting that they may contain more than one Raman peak. For further investigation, we demonstrate a more reliable fitting procedure than the commonly used least-squares method in the next paragraph, followed by our interpretation, including peak assignment, based on the fitting results.

**Figure 3:**
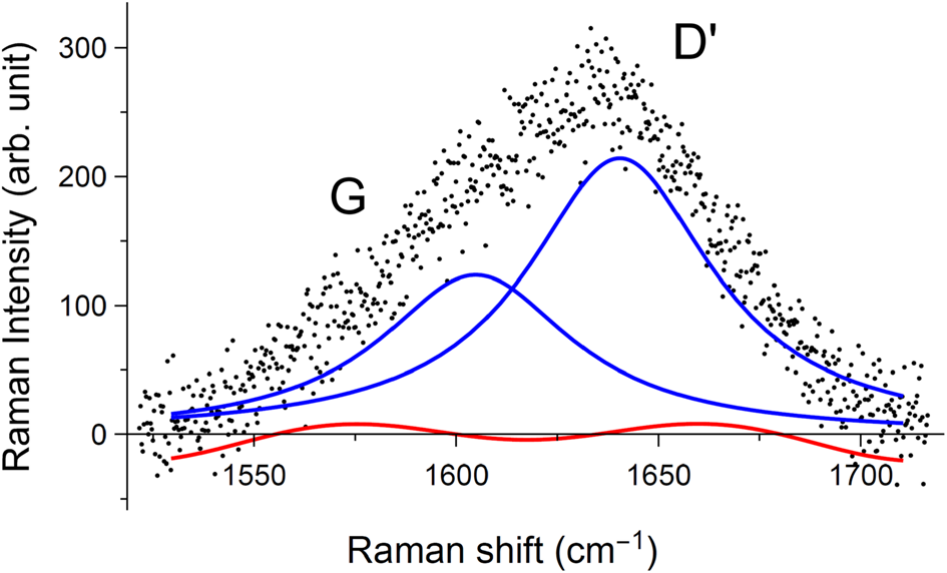
The optimal fitting for the spectrum of GO at 5.7 GPa in Fig. 2a. It consists of two Lorentzians and a Fourier series of two harmonics as the background. The labels on the peaks are the interpretations made after fitting.

In the range presented (1410–1680 cm^−1^), it is commonly considered that two modes, the G-mode at 1590 cm^−1^ and the defect-modified G-mode, D$^\prime$-mode at 1615 cm^−1^, can be observed in GO^24^. The Raman signal of $sp^3$ C–C from diamond (at about 1330 cm^−1^^25^, outside the range presented) overlaps with the D-mode of defected $sp^2$ carbon materials. There might be more modes in GO-M13 and GO-M13-CNT due to contributions from the CNT and M13 modes. Choosing the correct model, i.e. the number of peaks and the appropriate background, for these broad profiles is crucial to determine the fitting results. We take the spectrum at 5.7 GPa in Fig. 2a as an example of how different choices of model affect the results. A convincing conclusion requires an objective criterion to obtain the optimal fitting. We employed maximum likelihood estimation to fit the spectra with several models. The likelihood is the product of the probabilities of each residual, and maximising it is equivalent to minimising the sum of the squares of the residuals in least-squares fitting, and gives the same fitted parameter values (see SI). However, its interpretation is easier. A difference of $\Delta \ln L$ (*L* is the maximum likelihood) between two models gives odds of $e^{\Delta \ln L}$ in favour of the model with the higher $\ln L$. More parameters always give a higher *L*, and so the intuition of this is expressed by the Bayesian information criterion (BIC)^26^, which also applies a penalty for each parameter (see SI). We compared the BIC of different fittings. We present the optimal fitting of this spectrum in Fig. 3. It consists of two Lorentzians for the two modes and a Fourier series of two harmonics for the background, with the BIC value of 6555. The BIC value for two Lorentzians without background is 6705, for three Lorentzians without background is 6659, and for three Lorentzians with the same background as the optimal fitting is 6576. Sometimes the D$^{\prime }$ peak of the GO is fitted by a Fano lineshape. However, we found in our spectra that the improvement using that additional fitting parameter compared to a Lorentzian did not compensate for the extra parameter, with a higher BIC of 6561. A BIC higher by 6 is strong evidence against this fitting. The Raman frequencies (in cm^−1^) of all the fitted peaks in the spectra at close to ambient pressure of GO, GO-M13 and GO-M13-CNT are summarised in Table 1. These three spectra with peaks labelled are presented in the SI. The origins of all the peaks and the discrepancy of the peak positions across the three samples will be discussed in the following sections.

**Table 1:** The Raman frequencies (cm^−1^) of all the fitted peaks of GO, GO-M13 and GO-M13-CNT at close to ambient pressure are presented. The G-mode of GO-M13-CNT consists of the G$^+$ from CNTs and G-mode from GO, of very close frequency and about equal contribution. The G2 of GO-M13-CNTs consists of the G$^-$ from CNTs and modified G-mode by M13 from GO.

Sample	G1	G2	$\hbox {D}^\prime$
GO	1590	n/a	1615
GO-M13	1571	1532	1609
GO-M13-CNT	1592	1556	1613

**Figure 4:**
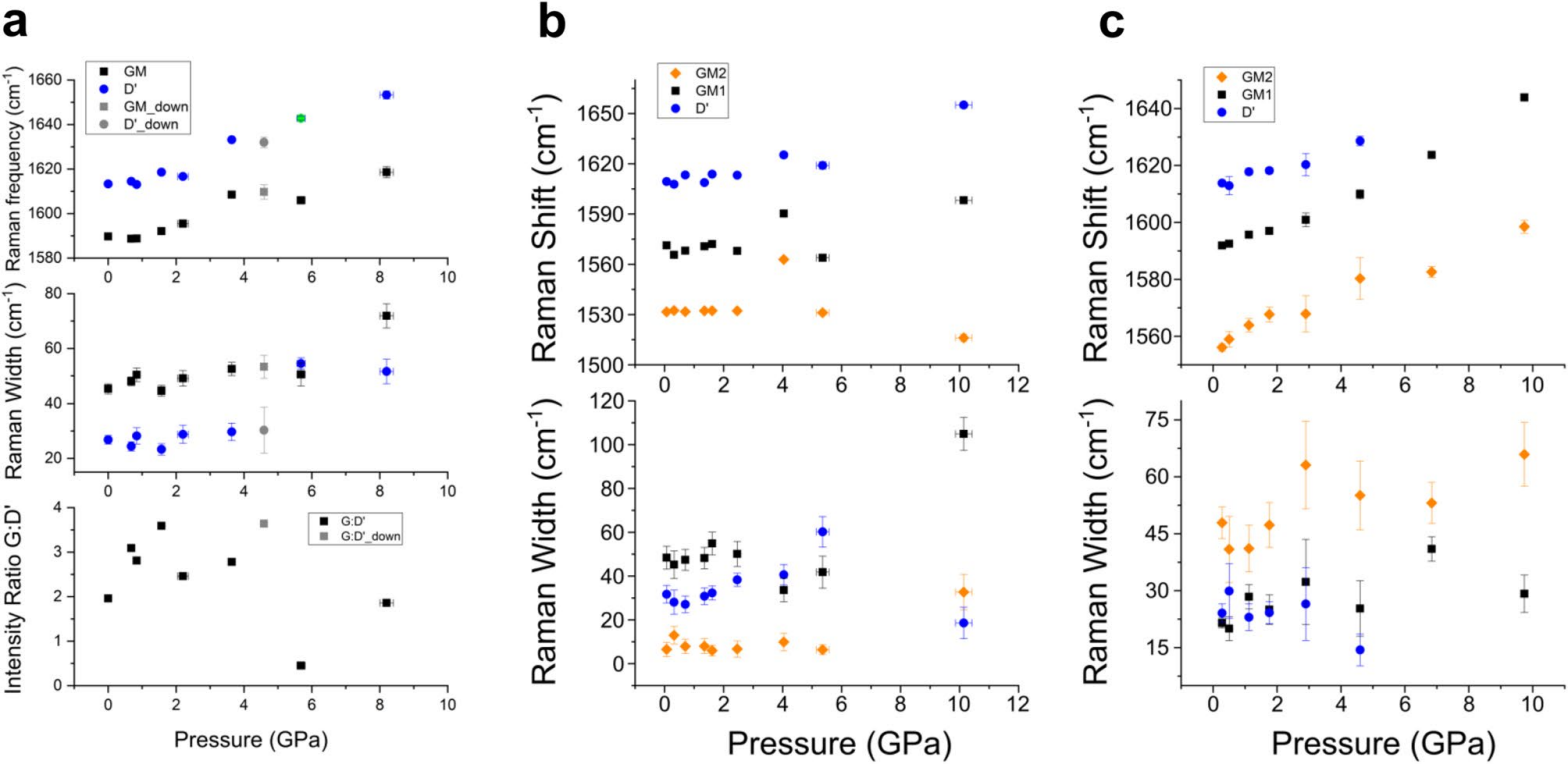
The pressure-dependence of the frequency and width of the G and D$^\prime$ modes in (**a**) GO, (**b**) GO-M13 and (**c**) GO-M13-CNT is presented. The intensity ratio between the G and D$^\prime$ modes in GO is also presented in (**a**). Error bars are shown where they exceed the size of data points. The data at 4.6 GPa (grey) were collected after the pressure was released from 8.2 GPa in (**a**).

We obtained the frequency, width and intensity of the Raman peaks by this fitting procedure for each spectrum, and plot them against pressure in Fig. 4a. The error bars in pressure, frequency and width of the Raman peaks are shown where they exceed the size of the data points. The uncertainties in pressure are from measurements using different ruby chips, indicating the departures from hydrostaticity. The errors in frequency and width are from the fittings and the resolution of the Raman system. The G-mode in GO comes from the $sp^2$ bonds in a graphene plane. The $sp^2$ bonds stiffen under pressure and therefore the G-mode frequencies are expected to upshift. The shift rate of its frequency with the effective in-plane force is expected to be consistent with that in graphite. However, it does not shift with pressure up to 0.8 GPa, suggesting that the in-plane force on a graphene plane changes very little within this pressure range. The other possibility is that the $sp^2$ bonds lose their anharmonicity, which is highly unlikely. The absence of an in-plane force shift with pressure below 0.8 GPa may be ascribed to the complicated behaviour of water in a confined space, which will be discussed later. The shift rate recovers to the graphite level in the range of about 1.0 to 3.6 GPa. There is no abrupt change of the width of the G and D$^\prime$ modes and of the intensity ratio between these two up to 3.6 GPa. The spectrum at 5.7 GPa evolves differently from the previous spectra obtained at lower pressures. If we assign the peak of a lower frequency to the G-mode, as we did in interpreting the data for lower pressures, the relative intensity of the G to D$^\prime$ mode drops significantly. It might be more reasonable to consider that it is the intensity of the D$^{\prime }$ that relatively increases, due to the previously reported redistribution of the functional groups, which can be considered as defects in graphene. This therefore determines the intensity of the D$^\prime$-mode, in GO under high pressure. The data point at 4.6 GPa was collected after the pressure was reduced from 8.2 GPa. Whereas the pressure calibration during pressure release can be very inaccurate, the spectrum at 4.6 GPa appears to be similar to those before 5.7 GPa.

### GO-M13

Raman spectra for GO-M13 as a function of pressure are shown in Fig. 2b. The signal to noise ratio is decreased compared to GO in Fig. 2a due to the reduced laser power from 5 mW to 1 mW on the sample to avoid burning. Both the G and D$^\prime$ modes from the GO are still present and there is visibly an additional peak at 1532 cm^−1^ (see Fig. 2b). We consider this additional peak to be a modified G-mode (G2) in GO, because there is no Raman peak from M13 near this frequency^5^.

We present the fitted frequencies and widths of the three modes in Fig. 4b. Apart from the clearly modified G2 at 1532 cm^−1^, the normal G-mode (G1) is downshifted, by about 20 cm^−1^ from pristine GO at ambient pressure. This may be due to charge transfer between GO and M13, or M13 softening the $sp^{2}$ bonds by disrupting the graphene plane. Both G1 and G2 shift very little (if at all) with pressure up to 2.5 GPa, again suggesting that the in-plane force applied on graphene planes does not shift with pressure. There is no abrupt change of the width and of the intensity ratio of the Raman modes from the lowest frequency to the highest up to 2.5 GPa. From 2.5 to 4.0 to 5.4 GPa, the intensity ratios of these three modes change significantly, from 1:25:22 to 1:8.6:9.2 to 1:8.5:13.

The most surprising observation in GO and GO-M13 is that the G-mode frequency of GO does not initially shift with pressure. As mentioned before, the G-mode of graphite shifts with hydrostatic pressure with a notable curvature due to the large anisotropy. The interlayer interaction of GO is even softer than graphite. This could decrease the shift rate substantially once at pressure, but should not reduce the initial shift rate. In Fig. 4a,b, there is no sign of an initial shift at all. Up to about 1 GPa, the G-mode frequency simply does not shift. We suggest that the complicated behaviour of water in a confined space is relevant here. First, bulk water is expected to transform from the liquid to the solid phase VI at 0.8 GPa^27,28^ and at about 2.5 GPa to transform further to phase VII at which pressure transmission is probably more efficient^29^. The phase transition of bulk water affects the hydrostaticity, which is indicated by measurements on ruby chips and shown as uncertainties in pressure. However, its effect on the G-mode frequency shift with pressure in GO should be similar to that in graphene or graphite. The non-monotonic shift at around 4 GPa observed in this work was not reported in graphene^30^ or graphite^16^. Therefore it is more reasonable to think that the outlier at about 4 GPa is due to structural change of GO rather than a phase transition of water. And the grey data point in Fig. 4a for releasing pressure after this structural change suggests that this transition is reversible. Second, we think that water enters the pores in Fig. 1h–k at a 10-micron-scale and transmits pressure as bulk. The pores are very large and would collapse under pressure if water was unable to enter and transmit pressure. Eventually pressure is applied to the nanostructures (schematics in Fig. 1e–g). The structure of water in a confined space, such as between GO layers (GO is hydrophilic^31^), is complicated and its phase diagram might be very different from bulk water^32^. Moreover, it was reported that water could be increasingly intercalated between GO layers and expand the interlayer spacing by 28–30% up to 1.3–1.5 GPa^33^. We think that the in-plane force on the C–C bonds does not shift with pressure until the bulk water solidifies, is more reasonably due to the behaviour of the pressure transmitting medium (water) in a confined space than the GO itself. We will further discuss about it after presenting the results of GO-M13-CNT.

### GO-M13-CNT

We now present the spectra of GO-M13-CNT under pressure in Fig. 2c. At the lowest pressure, the spectrum consists of three peaks at 1556, 1592 and 1613 cm^−1^. The CNTs sample that we used here has two G$^-$ peaks at 1528 and 1545 cm^−1^, corresponding to the (6, 4) and (6, 5) tubes (G$^-$ modes are vibrations along a tube circumference and therefore softened depending on the tube diameter). They are of lower intensity than their corresponding G$^+$ peaks, both at 1592 cm^−1^. Both G$^-$ peaks, together with a modified G-mode of GO similar to that in GO-M13, contribute to the broad peak at 1556 cm^−1^ (G2). The main peak at 1592 cm^−1^ consists of the G$^+$ of the CNTs and the main G-mode of GO at the same frequency (or at least very close). The peak at 1613 cm^−1^ can again be reasonably assigned to the D$^\prime$-mode of GO. Evidence of a structural change in GO-M13-CNT appears earlier at just 3.4 GPa. We previously performed a high pressure study on the same CNTs samples alone^34^. We reported that the (6, 5) tubes were in resonance at 568 nm. At 647 nm (what we use here), there were several radial breathing mode peaks from CNTs over the low frequency range (100–350 cm^−1^), without one dominating the spectrum (no tube is in resonance). This suggests that the contribution to the G-mode from CNTs of different chiralities in this work is roughly determined by their mass percentage—that is, (6, 5) tubes contribute more than 50%. It also ensures that the contribution of CNTs does not make it dominate the G-mode over GO.

We fit all the spectra of GO-M13-CNT and give the frequency and width of all the components in Fig. 4c. The shift rates of G1 (1592 cm^−1^ at the lowest pressure) and G2 (1556 cm^−1^ at the lowest pressure) are similar to those of the pure CNTs sample up to 0.9 GPa. The G-mode from GO is of higher intensity than the D$^\prime$-mode, and therefore makes a similar contribution as the G$^+$ of CNTs to the G-mode at 1592 cm^−1^. They probably shift at different rates and are much separated at 1.5 GPa, as indicated by the large errors of the width if we continue to fit the profile by one single peak. We see that unlike in GO and GO-M13, the G-mode of GO in GO-M13-CNT also shifts with pressure in this low-pressure range (where water is liquid phase in bulk). The other implication that the G-mode of GO shifts with pressure in GO-M13-CNT is that the D$^\prime$-mode shifts over 5 cm^−1^ up to 1 GPa while in GO and GO-M13 it shifts less than 5 cm^−1^ up to 2 GPa. Although what exactly determines the shift rate of the D$^\prime$-mode is unclear, the very different shift rate makes it convincing to conclude that the nanomechanics of GO-M13-CNT in water is different from GO and GO-M13. Carbon nanotubes of small diameters (0.7 nm for (6, 5) tubes) are hydrophilic at room temperature^35^. They do not prevent intercalation of water but might modify its structure or alignment, or affect the expansion of GO up to 1.3 GPa. The uncertainties in the width of peaks from 1.5 GPa are large, probably due to further splitting of these peaks due to their different shift rates with pressure. We therefore think that no further information can be reliably extracted.

### Further discussion

We consider why the G-modes of GO and GO-M13 do not shift with pressure. We note that it has been demonstrated experimentally that if graphene is attached to a stiffer substrate, the substrate protects the graphene from external compression^36^. We therefore suggest that if our GO is attached to the intercalated water between the GO layers, and if the intercalated water is stiffer than GO, this will protect the GO from external compression. This would be a novel phenomenon, different from, but related to, the previously reported intercalated water stiffening a nanostructure^37,38^. An alternative interpretation is linked to the observation that the G-mode frequency is reduced by about 20 cm^−1^ in GO-M13 at ambient pressure. Whatever it is that causes this, it may be enhanced with pressure so as to give the much-reduced initial pressure coefficients. However, we cannot make a definite interpretation without further evidence.

In this work, we focus on the mechanics of the material and not the biological activity. M13 is an extraordinary bio-component capable of resisting extreme conditions of temperature (inactivation temperature 80–${85}\,^{\circ }\hbox {C}$ for 10 min^39,40^) and pH (stable at pH ranging from 3 to 11^41^). Although many aspects of M13 have been deeply studied with numerous techniques, the effect of high pressure on its stability, related to its infectivity, has not been investigated yet in detail (certain bacteria, viral samples and nucleic acids (and indeed whole organisms—tardigrades are reported to survive 6 kbar^42^) have been reported to survive under pressure at a GPa range^43–45^). Potentially, it can preserve its biological activity (infectivity), however, for the fabrication of M13-based materials, the virus is usually subjected to chemical/genetic modification which reduces its natural infectivity. Moreover, some manufacturing approaches involving extreme conditions of pressure, pH and temperature as well as its templating with conductive materials, do not guarantee the preservation of its biological activity. For materials applications this is usually not crucial, given that the virus is exploited for its nanoscopic dimensions and for the broad range of functionalisation that it can manifest.

### Electrical conductivity

**Figure 5:**
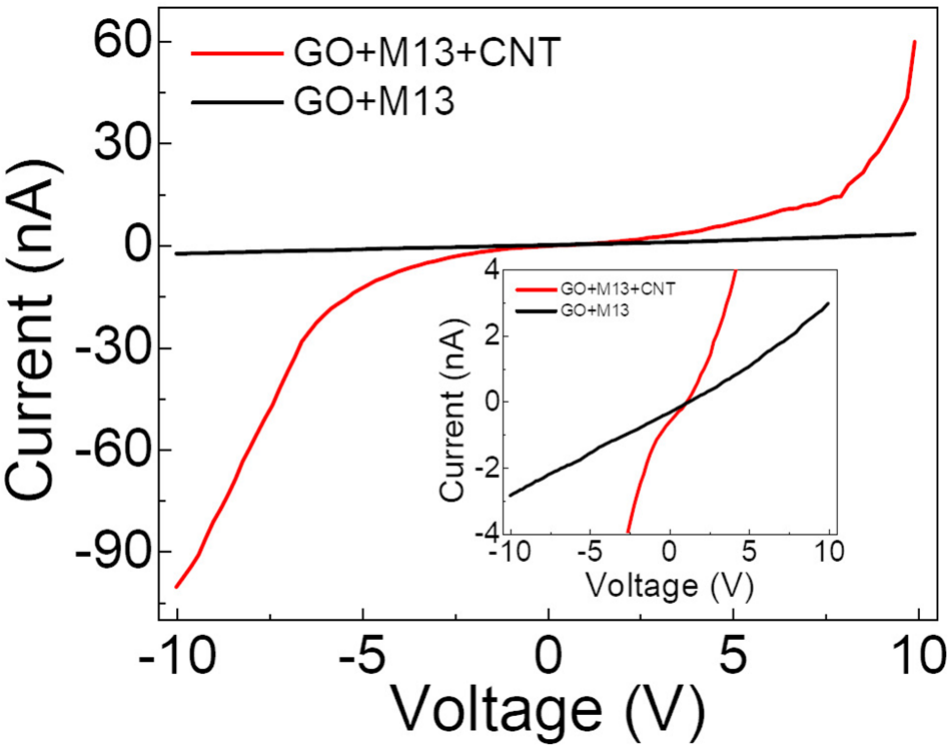
The I–V characterisation of the GO-M13-CNT aerogel, in comparison with GO-M13 of the same device size, showing a much improved electrical conductivity. Inset: the same data zoomed-in around 0 current to clearly show the values of GO-M13.

Finally, we present the I–V characteristic of the GO-M13 and GO-M13-CNT aerogel in Fig. 5. The electrical current of device GO-M13 is at the order of nA under a bias voltage of 10 V, which is the characteristic behaviour of an insulator. The current of device GO-M13-CNT is about 30 times larger than that of device GO-M13 at the same bias condition at 10 V, corresponding to an increase of conductance from 0.2 to 6 nS, indicating a much improved electrical conductivity with the presence of CNTs. The CNTs introduced to the GO-M13 aerogel provide conducting channels for the carriers, hence improving the conductivity of the composite. It is worth pointing out that the non-linearity in the I–V curve of the device GO-M13-CNT may be caused by the tunnelling of carriers between adjacent CNTs. The non-linearity may also be due to the non-ohmic contact between the gold electrode and our composites, which could be avoided by using matched metals as electrodes. The value can likely be further improved by composite optimisation, such as increasing the amount of CNTs, or selecting more suitable electrodes.

## Conclusions

By studying the evolution of the Raman peaks over the G-mode range with pressure, we find that in the GO the G-mode shifts very little with pressure up to 0.8 GPa, and then increases at a similar rate to graphite before a structural change (e.g. redistribution of functional groups) is observed at 5.7 GPa. This indicates that in the low pressure range, the in-plane force on the C–C bonds increases very little with pressure. We suggest that this is due to the complicated behaviour of water intercalated between the GO layers, rather than GO itself. In the GO-M13 composite, the non-shift range extends to 2.5 GPa and the structural change is observed earlier at 4.9 GPa. In strong contrast, the response to pressure of GO in GO-M13-CNT is more similar to that of graphite. This shows that with the same macroscopic morphology, and even with a similar nanostructure, the mechanics of slightly different composites can be very different due to intercalated water. The significant impact of the confined water is clearly evident but why it varies requires further investigation into the structure and properties of water in various nanostructures. After introducing CNTs during the process of self-assembly of GO and M13, the same porous sponge forms and a similar morphology is observed by SEM. With the presence of CNTs, water transmits pressure to the whole nanostructure as normal and the electrical conductivity is significantly improved, by about 30 times, giving the GO porous structure potential for applications where a good electrical conductivity is desired.

## Supplementary information

Supplementary information.
