# Supplementary material for: Nanomechanics of graphene oxide-bacteriophage based self-assembled porous composites
Source: Sci Rep. 2020 Sep 24;10:15618. doi: 10.1038/s41598-020-72372-1 (PMC7515913; doi:10.1038/s41598-020-72372-1)
Supplement: Supplementary file 1 — Supplementary information. [file 41598_2020_72372_MOESM1_ESM.pdf]

Nanomechanics of Graphene Oxide-bacteriophage based Self-assembled Porous Composites

Y. W. Sun[1,*], P. Passaretti[2], I. Hernandez[3,*], J. Gonzalez[3], W. Liu[4], F. Rodriguez[3], D. J. Dunstan[5], P. Goldberg Oppenheimer[6], and C. J. Humphreys[1,*]

[1]Queen Mary University of London, School of Engineering and Materials Science, London, E1 4NS, United Kingdom
[2]University of Birmingham, Institute of Cancer and Genomic Science, Birmingham, B15 2TT, United Kingdom
[3]Universidad de Cantabria, Departamento CITIMAC, Santander, 39005, Spain
[4]Zhejiang University, College of Information Science and Electronic Engineering, Hangzhou, 310027, China
[5]Queen Mary University of London, School of Physics and Astronomy, London, E1 4NS, United Kingdom
[6]University of Birmingham, School of Chemical Engineering, Birmingham, B15 2TT, United Kingdom
[*]yiwei.sun@qmul.ac.uk; ignacio.hernandez@unican.es; c.humphreys@qmul.ac.uk

ABSTRACT

Supplementary information. SEM images of all the samples. Maximum likelihood method and Bayesian information criterion. Raman spectra at close to ambient pressure of all the samples.

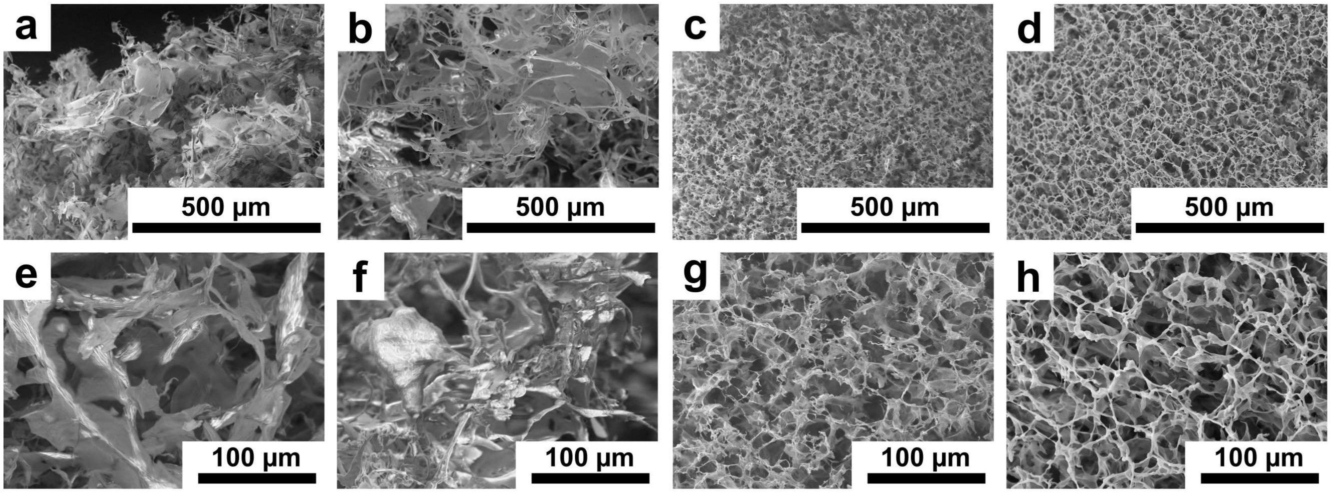

Figure 1. SEM images of a) GO, b) M13, c) GO-M13, d) GO-M13-CNTs, and again e) GO, f) M13, g) GO-M13, h) GO-M13-CNTs at a different magnification in random areas. All are secondary electron images.

Maximum likelihood method and Bayesian information criterion

The probability (we use Gaussian in this paper) of a residual with a magnitude of r is

$$P(r) = \frac{1}{\sqrt{2\pi\sigma^2}} e^{-\frac{r^2}{2\sigma^2}}, \tag{1}$$

where σ is the width of the Gaussian. The log-likelihood is

$$\ln L = \ln \prod_{i=1}^{n} P(r_i) = -n \ln \frac{1}{\sqrt{2\pi\sigma^2}} - \sum_{i=1}^{n} \frac{r_i^2}{2\sigma^2}. \tag{2}$$

So maximising the likelihood is equivalent to minimising the sum of the squares of the residuals in least-squares fitting. A difference of $\ln L$ between two models is unambiguously meaningful, enabling the Bayesian information criterion to apply a quantified penalty for each parameter as

$$BIC = k\ln(n) - 2\ln L, \tag{3}$$

where k is the number of parameters in a model, and n is the number of data points.

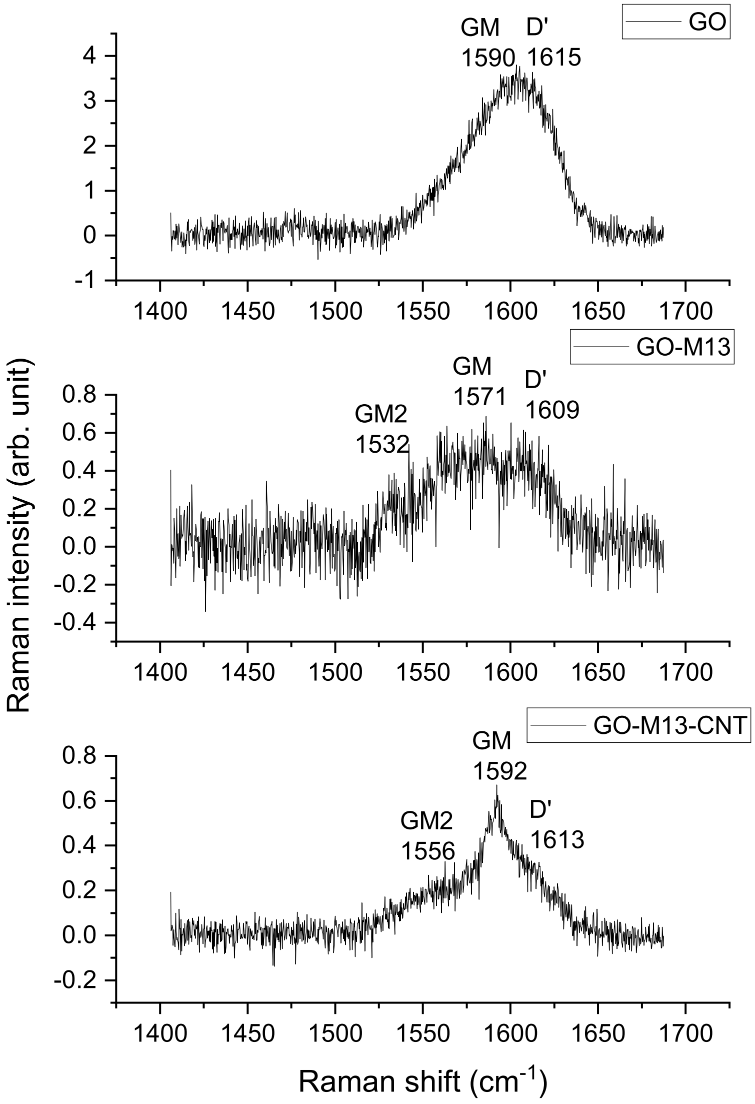

Figure 2. Raman spectra of GO, GO-M13 and GO-M13-CNT obtained at close to ambient pressure. We fitted the spectra employing Bayesian information criteria demonstrated in the main manuscript and SI. The labels on the peaks are the interpretations made after fitting.
